# Frailty Status and Transport Disadvantage: Comparison of Older Adults’ Travel Behaviours between Metropolitan, Suburban, and Rural Areas of Japan

**DOI:** 10.3390/ijerph17176367

**Published:** 2020-09-01

**Authors:** Takumi Abe, Akihiko Kitamura, Satoshi Seino, Yuri Yokoyama, Hidenori Amano, Yu Taniguchi, Mariko Nishi, Yu Nofuji, Tomoko Ikeuchi, Takemi Sugiyama, Shoji Shinkai

**Affiliations:** 1 Research Team for Social Participation and Community Health, Tokyo Metropolitan Institute of Gerontology, Tokyo 173-0015, Japan; kitamura@tmig.or.jp (A.K.); seino@tmig.or.jp (S.S.); yokoyama@tmig.or.jp (Y.Y.); amano@tmig.or.jp (H.A.); nishi@tmig.or.jp (M.N.); nofuji@tmig.or.jp (Y.N.); 2 Centre for Urban Transitions, Swinburne University of Technology, Melbourne, VIC 3122, Australia; tsugiyama@swin.edu.au; 3 Japan Society for the Promotion of Science, Tokyo 102-0083, Japan; 4 Center for Health and Environmental Risk Research, National Institute for Environmental Studies, Tsukuba 305-0053, Japan; taniguchi.yu@nies.go.jp; 5 Human Care Research Team, Tokyo Metropolitan Institute of Gerontology, Tokyo 173-0015, Japan; ikeuchi@tmig.or.jp; 6 Behavioural Epidemiology Laboratory, Baker Heart and Diabetes Institute, Melbourne, VIC 3004, Australia; 7 Kagawa Nutrition University, Sakado 350-0288, Japan; sshinkai@centm.center.tmig.or.jp

**Keywords:** ageing, car use, inequality, urban-rural differences, walking

## Abstract

This study examined differences in older adults’ travel behaviours by frailty status in metropolitan, suburban, and rural areas of Japan. Data were collected from 9104 older adults (73.5 ± 5.7 years; 51% women; 19% frail) living in metropolitan (n = 5032), suburban (n = 2853), and rural areas (n = 1219) of Japan. Participants reported if they walked, cycled, drove a car, rode a car as a passenger, and used public transportation (PT) once per week or more. A standardised questionnaire was used to assess frailty status. We conducted logistic regression analysis to calculate the odds ratios of using each travel mode by frailty status stratified by locality. Relative to non-frail participants, frail participants were less likely to walk and drive a car in all three areas. Frail participants had significantly higher odds of being a car passenger in the suburban (OR = 1.73 (95% CI: 1.32, 2.25)) and rural areas (OR = 1.61 (1.10, 2.35)) but not in the metropolitan area (OR = 1.08 (0.87, 1.33)). This study found that frail older adults living in suburban and rural areas tended to rely more on cars driven by someone else, suggesting that transport disadvantage is more pronounced in suburban and rural areas than in metropolitan areas.

## 1. Introduction

Transport disadvantage is typically defined as the inability to travel when and where one needs without difficulty [1]. Such disadvantage is associated with poorer quality of life and well-being [2], as it can deprive people of opportunities to engage in meaningful social and community activities [3]. Transport disadvantage among older adults is a contemporary issue, since the population is ageing rapidly, particularly in developed countries [4]. The effect of transport disadvantage may be magnified in older adults who tend to have a limited capacity to move and begin to give up driving [5]. Addressing transport disadvantage and reducing inequities among older adults is an important step towards creating an age-friendly society [4]. Inability to travel can result in social exclusion, which is also salient to older adults, who tend to have a limited social network [6]. Since social exclusion among older adults is common and can have implications on their health and well-being [7], it is important to address transport disadvantage among older adults to ensure they have adequate transport means to access necessary goods and services and engage in social activities. Transport disadvantage is a multi-dimensional construct, consisting of individual-level (e.g., levels of physical function) and contextual-level factors (e.g., access to shops and services, availability of public transportation service) [3]. For instance, levels of disadvantage in one’s environment can vary depending on a person’s individual-level mobility (availability of private motor vehicles or levels of physical function) [8].

Frail older adults are more likely to suffer from transport disadvantage [3]. Frailty is a condition where a person’s psychological well-being, physical function, and the ability to cope with stressors are reduced [9]. It is important to note that frailty is a precursor to disability [10]. Since disability is difficult to reverse at older age, frailty has been considered as a window of opportunity to prevent disability. For instance, promoting physical activity and participation in social activities among frail older adults can help them to slow down the process towards disability [11,12]. Transport disadvantage is relevant in this context. Frail older adults who are disadvantaged in transport may be at greater risk of losing functional capacity than those who are able to travel with multiple transport options, since the former may lack opportunities to be physically active and be connected to the local community. 

It is possible that the level of transport disadvantage frail older adults experience can differ between the locations where they live. For instance, metropolitan areas tend to have more well-developed, easier-to-use transport infrastructure than suburban and rural areas. Thus, transport disadvantage among frail older adults may be exacerbated in regional areas. However, it is unknown to what extent travel mode choice differs by frailty status in different localities. A better understanding of where transport disadvantage is more pronounced is important to inform strategies and policies to ensure equitable transportation. 

This study examined differences in travel behaviours by frailty status and whether such differences are equally present in metropolitan, suburban, and rural areas of Japan.

## 2. Methods

### 2.1. Data Sources and Participants

This study combined data obtained from three localities in Japan: Ota ward, Tokyo (population density: 11,814 persons/km^2^ in 2016); Hatoyama town, Saitama prefecture (population density: 541 persons/km^2^ in 2018); and Kusatsu town, Gunma prefecture (population density: 132 persons/km^2^ in 2014). They were categorised as metropolitan, suburban, and rural, respectively, based on the classification of the Japanese population census [13]. 

A description of data collection in each locality has been reported elsewhere [14,15,16]. Briefly, a mail survey was conducted in Ota ward in 2016 as the baseline assessment for a community-wide intervention trial. A total of 15,500 older adults aged 65 or older without disability were randomly selected for recruitment from three intervention sites (8000 participants) and 15 control sites (7500 participants), each of which was stratified by age and gender. Given that participants in the intervention sites were considered to be more representative of the population than those in the control sites (due to the larger sample size per site), the present study chose 6009 respondents living in the intervention sites. The study in Hatoyama town was conducted in 2018. A self-reported questionnaire was mailed to 5150 older adults aged 65 or older without disability, of which 3335 consented to participate and returned the questionnaire. For the study in Kusatsu town, we conducted a survey targeting 1977 older adults aged 65 or older in 2014 and received responses from 1693 without disability. In this study, people without disability were defined as those who did not receive any support or care for their daily activities [17]. Each study was approved by the ethical committee of the Tokyo Metropolitan Institute of Gerontology.

### 2.2. Frailty Assessment

Frailty status was assessed by the “Kaigo-Yobo” (disability prevention) Checklist (KYCL), which was composed of 15 items regarding the physical, nutritional, and social aspects of frailty [16] (Supplementary Table S1). The KYCL has been found a valid and reliable instrument for assessing frailty status [18]. The score ranges from 0 to 15, with a higher score indicating poorer health status. Those scoring 4 or above were classified as frail, and the classification is commonly used in previous studies [18,19,20,21].

### 2.3. Travel Modes

Participants’ usual modes of travel were assessed by asking whether they used each of the following modes once per week or more: walking; cycling; car use as a driver; car use as a passenger; and use of public transportation (PT). Participants responded yes or no for each travel mode.

### 2.4. Statistical Analyses

Logistic regression analysis was performed to examine the association of the use of each travel mode with frailty status for each locality, adjusting for age, gender, living arrangement (living alone or not), and medical history (stroke, bone and joint disease). Analysis also adjusted for locality when the whole sample was examined. In order to check if localities differed in their associations of travel mode with frailty, we examined interaction between locality and frailty by entering interaction terms as an independent variable (metropolitan × frailty as the reference group). All analyses were computed using STATA 15.1 (StataCorp, College Station, TX, USA). The statistical significance level was set at 0.05.

## 3. Results

### 3.1. Sample Characteristics

After excluding those with missing data on key variables, the final sample size for analysis was 9104. Table 1 shows characteristics of participants by frailty status. Walking was the most common travel modes in both non-frail and frail participants. In all travel modes except car use as a passenger, non-frail participants had higher prevalence than frail participants. Table 2 shows the prevalence of travel mode by locality and frailty status. Chi-square tests showed significant differences in all travel modes between localities both among non-frail and frail participants (*p* < 0.001).

### 3.2. Differences between Non-Frail and Frail Participants in Travel Modes

Table 3 shows odds ratios of using each travel mode by frail participants relative to non-frail participants for the whole sample and in each locality. For the total sample, frail participants were less likely to walk, cycle, use a car as a driver, and use PT, and were more likely to use a car as a passenger, compared to non-frail older adults. For locality-specific analyses, frail participants were found less likely to walk and drive a car compared to non-frail participants in all three localities. Relative to non-frail participants, frail participants had a significantly lower odds ratio of cycling in the metropolitan area and a significantly lower odds ratio of PT use in the metropolitan and suburban areas. Frail participants living in suburban and rural areas were more likely to use car as a passenger than non-frail participants.

Table 3 also shows the interaction terms between locality and frailty. There was no significant interaction of frailty status and localities for walking and cycling, indicating no effect modification by locality (i.e., the difference in travel mode use by frailty status did not vary by locality). A significant interaction was observed for car driving in the rural area, car use as a passenger in the suburban and rural areas, and PT use in the rural area, suggesting effect modification for the respective areas relative to the metropolitan area.

All models adjusted for age, gender, living arrangement, and medical history (stroke, bone and joint disease). The analysis for total sample was further adjusted for locality.

## 4. Discussion

This study analysed data on travel modes of non-frail and frail older adults living in metropolitan, suburban, and rural areas of Japan. Our results showed that frail older adults in general were less likely to walk, cycle, drive a car, and use PT, and more likely to be a car passenger compared to those without frailty. This study found that such disparities in travel modes (car driving, car passenger, and PT use) for frail participants were not homogeneously distributed in different areas. It was found that frail participants were more likely to be car passengers in suburban and rural areas, but this was not the case in metropolitan areas. (Note that a significant interaction was found for car use as a passenger by frail participants in suburban and rural areas relative to metropolitan areas.) These findings suggest that frail older adults in suburban and rural areas rely more on cars driven by someone else, indicating that they may not be independent with regard to their travel. In contrast, frail older adults in metropolitan areas are not significantly different from non-frail older adults in terms of car use as a passenger. It is possible that those living in metropolitan areas do not have to travel long distance for regular needs (e.g., shopping, attending services, participating in social activities), which may be done without relying on a lift by someone regardless of frailty status. Even when destinations are not nearby, PT may be available to access such places in metropolitan areas. In contrast, travel distances for such destinations may be longer in suburban and rural areas. Without an adequate PT network, they could be hard to reach for older adults with frailty by themselves. It is also possible that frail older adults in suburban and rural areas are more likely to be supported by relatives and friends in their local community than those in metropolitan areas. It has been shown that older adults living in rural areas tend to have a higher level of social engagement with others compared to those in metropolitan areas [14]. Although relatives or friends may be available to meet travel needs of frail older adults in suburban and rural areas, that does not necessarily mean that they can travel whenever they need. Their travel can be restricted depending on the schedule of those who can assist them. 

Providing alternative transport services such as on-demand mobility and ride-share services in suburban and rural areas, where public transport service is not easily accessible, would mitigate transport disadvantage. Considering an increasing commercial interest in new urban mobility, establishing a mechanism to enable “public-private partnership” appears to be a promising strategy to promote new mobility services [22]. Encouraging compact, transport-oriented development targeting older adults may be a long-term solution from a planning perspective, as such development can not only facilitate active living (e.g., walking to local shops and services) but also generate concentrated travel demand, which may make it easier to provide diverse transport options. A concerted effort between transport and planning sectors is needed to address transport disadvantage among older adults.

Rural areas also differed in the odds ratios of car driving and PT use by frail participants, in comparison to metropolitan areas. It is unknown why the odds ratio of driving by frail older adults in the rural area was lower than that in the metropolitan area. It could be argued that roads in rural areas are less congested and thus easier to drive on for those with frailty, compared to busier roads in metropolitan areas. There may be other factors that discourage frail older adults from driving in rural areas (e.g., poor lighting, the presence of others who can drive for them). It should be noted that car driving is beneficial for older adults to a certain degree, as it is known to be associated with more physical activity and more opportunities for social engagement [23,24]. Given that prolonged car use, a common sedentary behaviour, is known to be related to greater cardiometabolic risk, future studies need to assess to what extent car use among older adults contributes to better health through additional opportunities for activities or to ill health due to prolonged sitting. With regard to PT use, the results could suggest that frail older adults in rural areas were not as disadvantaged in using PT than non-frail older adults. However, as shown in Table 2, the prevalence of PT use was lower than 20% in rural areas for both non-frail and frail participants. The findings appear to reflect that PT is not a common mode of travel in rural areas, irrespective of frailty status. 

The odds ratios of walking and cycling by frail participants did not differ significantly between the three areas (non-significant interaction). It was anticipated that characteristics of suburban and rural areas such as poor provision of sidewalk and bicycle lane, long distance to destinations, and inconvenient PT systems would make it harder for frail participants in suburban and rural areas to walk in their local area. However, we found that frail participants engaged in less walking than non-frail participants in a roughly equal manner in all three areas. It is possible that our measure of travel modes (weekly use) may not be sensitive enough to pick up the differences of frail and non-frail participants between the three areas. Detailed measures of travel modes (frequency and duration, potentially using the travel diary format) are needed to address this issue. For cycling, there were large differences in the prevalence of cycling between metropolitan, suburban, and rural areas (Table 2). The number of participants who reported cycling was small, particularly in rural areas, which may have contributed to a lack of significant interactions. A study conducted in a different rural area of Japan reported that 28.3% of older adults were cyclists [23]. Compared to that study, the prevalence of cyclists in the rural area in this study was much lower (≈2%). It is not totally clear why there was such a large difference between the studies. One possibility is the response rate, which was 86% in this study (rural area) and 17% in the other Japanese study [23]. Our study may have included those who were not disabled but were not confident enough about cycling. Nevertheless, it is notable that the odds ratio of cycling by frail older adults relative to non-frail older adults (0.74) was larger than that of walking (0.51), car driving (0.48), and PT use (0.50) as shown in Table 3 (results for the total sample). Although all the coefficients were significant, the difference between non-frail and frail participants was smaller in bicycle use than in the other modes. This suggests that cycling may be a suitable mode of travel for frail older adults in Japan. Cycling may be promoted as an alternative transport mode in suburban and rural areas to mitigate transport disadvantage. Typically, creating safe environments is of primary importance to promote cycling. However, our study found high prevalence of bicycle use in the metropolitan area (with much motor traffic) and lower prevalence in the suburban and rural areas. Research is warranted to better understand factors that facilitate or hinder cycling among older adults in the context of Japan. 

The strengths of this study include the use of large datasets obtained from diverse areas of Japan. We identified the weekly use of five travel modes, which are likely to cover typical travel modes that older adults use. However, there are some limitations. First, we used a single item to examine the use of each travel mode. Although this is likely to be easy to recall for older adults, more detailed measures of travel behaviours are needed to further understand how frail residents are disadvantaged in transport and how such disparities differ by localities. Second, although we used a validated cut-off point that has been established in existing studies [18], there is a possibility of misclassifying participants. Future studies may examine the relationships using an alternative measure to assess participants’ frailty status [25]. Third, our data were obtained from three Japanese localities. In particular, the metropolitan area was a central district of Tokyo, where there is a well-developed network of public transport. The contrast in terms of access to public transport between the metropolitan and suburban/rural areas can be quite large. Thus, our findings from which may not be generalisable to regions within which there are limited differences in access to PT and in residents’ travel behaviours. Fourth, we could not adjust for some potential confounders, in particular participants’ socio-economic status, due to different formats used to elicit information from three localities. Lastly, data were collected in different years in three study areas. Given that the population is decreasing in many rural areas in Japan, the timing of data collection may have influenced our results.

## 5. Conclusions

Frail older adults are generally disadvantaged compared to non-frail older adults in transport options. This study added that transport disadvantage appeared to be more pronounced in suburban and rural areas, where frail older adults had to rely more on someone else’s car for regular travel. Considering that “ageing in place” is a key policy strategy in ageing countries such as Japan [26], it is important to ensure that frail residents have adequate means of transport to meet their needs and wants. Introducing alternative transport modes in suburban and rural areas (e.g., rideshare, on-demand transport) may be a potential initiative to mitigate such disparities in transportation.

## Figures and Tables

**Table 1:** Characteristics of participants by frailty status.

		Non-Frail (n = 7390)	Frail (n = 1714)	Total (n = 9104)
Age	mean ± SD	73.1 ± 5.5	75.3 ± 6.1	73.5 ± 5.7
Gender	% women	52.5	45.5	51.1
Living arrangement	% living alone	14.7	17.0	15.2
Medical history	% with stroke	4.3	9.5	5.2
	% with bone and joint disease	19.7	31.6	22.0
Travel mode ^a^	% walkers	79.1	67.0	76.9
	% cyclists	31.6	30.9	31.5
	% car drivers	47.3	28.7	43.8
	% car passengers	17.4	20.0	17.9
	% PT users^a^	55.3	46.9	53.7

^a^ Using each travel mode once per week or more.

**Table 2:** Travel modes by locality and frailty status.

	Metropolitan		Suburban		Rural	
Travel Mode ^a^	Non-Frail (n = 3904)	Frail (n = 1128)	Non-Frail (n = 2451)	Frail (n = 402)	Non-Frail (n = 1035)	Frail (n = 184)
% walkers	85.7	73.7	71.0	49.0	73.6	65.2
% cyclists	51.3	43.3	12.5	10.0	2.5	1.1
% car drivers	29.9	20.4	72.1	52.2	54.3	27.7
% car passengers	12.1	11.6	22.9	35.6	24.6	37.0
% PT users	74.9	58.7	39.2	26.1	19.5	19.6

^a^ Using each travel mode once per week or more.

**Table 3:** Odds ratios of using each travel mode by frail participants (reference: non-frail) for the whole sample and stratified by locality.

Travel Mode	Metropolitan		Suburban		Rural		Total
	OR (95% CI)	Interaction	OR (95% CI)	Interaction ^1^	OR (95% CI)	Interaction ^1^	OR (95% CI)
Walking	0.47 (0.40, 0.55) **	Reference	0.38 (0.30, 0.47) **	*p* = 0.157	0.57 (0.40, 0.80) *	*p* = 0.076	0.51 (0.45, 0.57) **
Cycling	0.73 (0.64, 0.84) **	Reference	0.80 (0.56, 1.14)	*p* = 0.497	0.55 (0.13, 2.40)	*p* = 0.586	0.74 (0.65, 0.84) **
Car driving	0.54 (0.46, 0.65) **	Reference	0.46 (0.35, 0.61) **	*p* = 0.216	0.33 (0.22, 0.49) **	*p* = 0.026	0.48 (0.42, 0.55) **
Car passenger	1.08 (0.87, 1.33)	Reference	1.73 (1.32, 2.25) **	*p* < 0.001	1.61 (1.10, 2.35) *	*p* = 0.008	1.36 (1.17, 1.58) **
PT use	0.47 (0.41, 0.54) **	Reference	0.52 (0.40, 0.66) **	*p* = 0.628	0.83 (0.54, 1.26)	*p* = 0.002	0.50 (0.44, 0.56) **

* *p* < 0.05, ** *p* < 0.01, PT: public transportation. ^1^ Significance of interaction terms between locality and frailty (reference: metropolitan area).

## Supplementary Materials

The following are available online at https://www.mdpi.com/1660-4601/17/17/6367/s1, Table S1: Kaigo-Yobo Checklist.
