# Supplementary material for: Frailty Status and Transport Disadvantage: Comparison of Older Adults’ Travel Behaviours between Metropolitan, Suburban, and Rural Areas of Japan
Source: Int J Environ Res Public Health. 2020 Sep 1;17(17):6367. doi: 10.3390/ijerph17176367 (PMC7504364; doi:10.3390/ijerph17176367)
Supplement: Supplementary file 1 [file ijerph-17-06367-s001.zip › Table - 3.DOCX]

Table 3. Odds ratios of using each travel mode by frail participants (reference: no-frail) for the whole sample and stratified by locality

| Travel mode | Metropolitan | | Suburban | | Rural | | Total |
| --- | --- | --- | --- | --- | --- | --- | --- |
|  | OR (95% CI) | Interaction | OR (95% CI) | Interaction^1^ | OR (95% CI) | Interaction^1^ | OR (95% CI) |
| Walking | 0.47 (0.40, 0.55)** | Reference | 0.38 (0.30, 0.47)** | p=.157 | 0.57 (0.40, 0.80)* | p=.076 | 0.51 (0.45, 0.57)** |
| Cycling | 0.73 (0.64, 0.84)** | Reference | 0.80 (0.56, 1.14) | p=.497 | 0.55 (0.13, 2.40) | p=.586 | 0.74 (0.65, 0.84)** |
| Car driving | 0.54 (0.46, 0.65)** | Reference | 0.46 (0.35, 0.61)** | p=.216 | 0.33 (0.22, 0.49)** | p=.026 | 0.48 (0.42, 0.55)** |
| Car passenger | 1.08 (0.87, 1.33) | Reference | 1.73 (1.32, 2.25)** | p < .001 | 1.61 (1.10, 2.35)* | p=.008 | 1.36 (1.17, 1.58)** |
| PT use | 0.47 (0.41, 0.54)** | Reference | 0.52 (0.40, 0.66)** | p=.628 | 0.83 (0.54, 1.26) | p=.002 | 0.50 (0.44, 0.56)** |

* p < 0.05, ** p < 0.01, PT: public transportation

^1^ Significance of interaction terms between locality and frailty (reference: metropolitan area)

All models adjusted for age, gender, living arrangement, and medical history (stroke, bone and joint disease). The analysis for total was further adjusted for locality.
